# Effect of treatment with a JAK2-selective inhibitor, fedratinib, on bone marrow fibrosis in patients with myelofibrosis

**DOI:** 10.1186/s12967-015-0644-4

**Published:** 2015-09-10

**Authors:** Catriona Jamieson, Robert Hasserjian, Jason Gotlib, Jorge Cortes, Richard Stone, Moshe Talpaz, Jürgen Thiele, Scott Rodig, Olga Pozdnyakova

**Affiliations:** Moores UC San Diego Cancer Centre, 3855 Health Sciences Drive, La Jolla, CA 92093-0820 USA; Department of Pathology, Massachusetts General Hospital, 55 Fruit Street, Boston, MA 02114 USA; Division of Hematology, Stanford University School of Medicine/Stanford Cancer Institute, 875 Blake Wilbur Drive, Room 2324, Stanford, CA 94305 USA; Division of Cancer Medicine, Department of Leukemia, University of Texas MD Anderson Cancer Center, Faculty Center Building on Floors 3 and 4, 1515 Holcombe Blvd., Houston, TX 77030 USA; Dana-Farber Cancer Institute, 450 Brookline Avenue, Boston, MA 02215 USA; The University of Michigan Hospital and Health Systems, Comprehensive Cancer Center, 1500 East Medical Center Drive, Ann Arbor, MI 48109 USA; Institute of Pathology, University of Cologne, Kerpener Str. 62, 50924 Cologne, Germany; Department of Pathology, Brigham and Women’s Hospital, 75 Francis Street, Boston, MA 02115 USA

**Keywords:** Fibrosis, Janus kinase inhibitor, Myelofibrosis, Myeloproliferative neoplasms, Philadelphia chromosome-negative

## Abstract

**Background:**

Progressive bone marrow fibrosis (BMF) is a cardinal feature of many myeloproliferative neoplasms (MPNs) and there is a documented association between the severity of BMF and overall prognosis. We conducted an exploratory analysis of sequential BMF data from two phase I studies of long-term treatment with the Janus kinase 2 (*JAK2*) inhibitor fedratinib in patients with myelofibrosis.

**Methods:**

Bone marrow samples were obtained at baseline and after every six cycles (24 weeks) of daily fedratinib treatment. Fibrosis was centrally assessed by three independent haematopathologists, who were blinded to the patients’ data, and graded according to European Consensus Myelofibrosis Grading Criteria. The analysis population comprised patients with a baseline BMF grade ≥1, and at least one post-baseline BMF grade assessment. Changes in BMF grade compared with baseline were classified as improvement (≥1 grade reduction), stabilisation (no change in any baseline BMF grade <3) or worsening (≥1 grade increase).

**Results:**

Twenty-one patients were included in the analysis. A total of 153 bone marrow samples were analysed. Improvement or stabilisation of BMF from baseline was recorded in 15 of 18 (83 %) evaluable patients at cycle 6 and in four of nine (44 %) evaluable patients at cycle 30. Two patients achieved resolution of their BMF (grade = 0) by cycle 12.

**Conclusions:**

This exploratory analysis indicates that improvement or even resolution of BMF may be achievable with *JAK2* inhibitor therapy in some patients with MPNs and myelofibrosis.

**Electronic supplementary material:**

The online version of this article (doi:10.1186/s12967-015-0644-4) contains supplementary material, which is available to authorized users.

## Background

Myelofibrosis is a feature of several Philadelphia-chromosome negative myeloproliferative neoplasms (MPNs), such as primary myelofibrosis (PMF), essential thrombocythaemia (ET) and polycythaemia vera (PV) [1]. Myelofibrosis can either be present at baseline, as seen in patients with PMF, or develop upon disease progression in patients with ET and PV [2, 3]. This group of MPNs is frequently associated with Janus kinase (*JAK*)2 V617F, *MPL* W515 L/K or exon 9 *CALR* gene mutations that have been reported to activate *JAK2*/signal transducer and activator of transcription (*STAT*) signalling [4–9]. Clinically, the presence of myelofibrosis is associated with splenomegaly, peripheral blood cytopaenias and constitutional symptoms including fatigue, night sweats and fever [3, 10, 11]. Several studies have demonstrated associations between the degree of bone marrow fibrosis (BMF), as assessed by morphological analysis, peripheral blood count abnormalities, splenomegaly and overall prognosis in patients with myelofibrosis [12–16].

The only potentially curative treatment for patients with myelofibrosis is allogeneic haematopoietic cell transplantation, which can result in a rapid reduction in BMF [17]. However, advanced age and co-morbidities preclude this as an option for most patients [18, 19]. Conventional clinical management has therefore focused on supportive therapies that palliate myelofibrosis symptoms, but appear to have no significant effect on BMF [12, 16]. Recent data from an open-label study and a case report suggest that long-term therapy with the *JAK*1/2 inhibitor ruxolitinib may improve or stabilise the progression of BMF in a proportion of patients with myelofibrosis [20, 21].

Fedratinib (SAR302503; TG101348) is a *JAK2*-selective inhibitor that was developed as a treatment for MPNs based on pre-clinical data that showed it caused a reduction in *JAK2* mutant allele burden and BMF in murine models of myeloproliferative disease [22, 23]. Clinical evaluation of fedratinib began in 2008 and results of phase I–III studies demonstrated clinical benefit as evidenced by reductions in splenomegaly and symptom burden in patients with myelofibrosis [24–26]. The clinical evaluation of fedratinib also included prospective assessment of the effects of long-term *JAK2* inhibition on BMF status. Although clinical development of fedratinib was discontinued in November 2013 (due to a few reports of treatment-emergent encephalopathy, resembling Wernicke’s), the effects of *JAK2* inhibition on BMF may be relevant for predicting the long-term efficacy of other *JAK2* inhibitors. We therefore report the results of an exploratory analysis of sequential BMF data from two phase I studies of long-term fedratinib treatment in patients with myelofibrosis.

## Methods

### Patients, studies and treatment

Patients with PMF, post-ET MF and post-PV MF participated in a phase I dose-escalation study (TED12037; Clinicaltrials.gov identifier: NCT00631462) of daily oral fedratinib [24] and its long-term extension study TED12015 (NCT00724334). Patients were eligible for entry into TED12015 if they had tolerated fedratinib therapy and had achieved clinical benefit, defined as stable disease, clinical improvement, partial remission or complete remission in accordance with the International Working Group for Myelofibrosis Research and Treatment (IWG-MRT) response criteria [27, 28], following six 4-week cycles of fedratinib in TED12037. Patients entered TED12015 immediately upon completion of TED12037, with no gap in treatment, and continued on the same daily dose of fedratinib in consecutive 4-week cycles. Patients were permitted to remain on treatment for as long as they continued to derive clinical benefit. Treatment was discontinued in the event of symptomatic or disease progression, unacceptable toxicity, or patient non-compliance/withdrawal of consent. In the absence of disease progression or unacceptable toxicity, the dose of fedratinib could be escalated up to the maximum tolerated dose of 680 mg/day.

### Assessment of bone marrow fibrosis

Bone marrow trephine biopsies were obtained at baseline and after six cycles (24 weeks) of treatment in TED12037. Repeat biopsies continued to be obtained every six cycles for the duration of TED12015. Biopsy sections were stained with haematoxylin and eosin, reticulin and Masson’s trichrome in order to allow grading of BMF on a scale from 0 to 3 using the European Consensus Myelofibrosis Grading Criteria. A description of the grading system is shown in Table 1 [29]. Staining was performed by the local laboratory at each of the six study sites.

**Table 1:** Grading criteria for bone marrow fibrosis [29]

Grading	Description
0	Scattered linear reticulin with no intersections corresponding to normal bone marrow
1	Loose network of reticulin with many intersections, especially in perivascular areas
2	Diffuse and dense increase in reticulin with extensive intersections, occasionally with only focal bundles of collagen and/or focal osteosclerosis
3	Diffuse and dense increase in reticulin with extensive intersections with coarse bundles of collagen, often associated with significant osteosclerosis

Bone marrow fibrosis is assessed in areas of haematopoietic cellularity

Stained sections were analysed and graded centrally by three independent haematopathologists using a predefined charter. Reviewers were blinded to subject demographics (except for patient age and gender), treatment time point, clinical response, clinical history and the local pathology results. A consensus on fibrosis grading required agreement by at least two pathologists; if all three reviewers disagreed discrepancies were resolved by open discussion followed by a consensus fibrosis score. All bone marrow samples were from patients with a baseline BMF grade of ≥1 in TED12037 and with at least one post-baseline BMF grade assessment (in either study). Changes in BMF grade, after every six cycles of treatment, were compared with the TED12037 baseline and classified as: improvement (≥1 grade reduction), stabilisation (no change in any baseline grade <3), persistent grade 3, or worsening (≥1 grade increase).

### Other clinical assessments

Splenomegaly was assessed by palpation (cm below the left costal margin) during physical examination, at baseline and every subsequent treatment cycle. Haematology parameters (including haemoglobin and white blood cell [WBC] count) were also assessed at baseline and every subsequent cycle.

### Role of the funding source

The study sponsor participated in the design of the clinical studies and in the collection of the fibrosis data. The sponsor played no role in the interpretation of the fibrosis data presented in this paper. The sponsor funded medical writing assistance and reviewed the final draft for study-parameter related accuracy. The final decision to publish the paper was made solely by the authors.

## Results

### Patients

Of the 59 patients who were treated with fedratinib in TED12037, 15 patients discontinued treatment before the completion of six cycles (due to AEs [n = 6], investigator decision/intercurrent illness [n = 3], or withdrawal of consent [n = 6]). One additional patient opted not to enter the extension phase study. Therefore, 43 of the 59 patients (73 %) enrolled in TED12015 and continued on fedratinib treatment. Twenty-one (36 %) of these patients were eligible for inclusion in the BMF assessment. The main reasons for exclusion from the BMF assessment were missing baseline samples and absence of documented patient consent. Demographics and baseline disease characteristics of the BMF analysis population (n = 21) and the all-treated population (n = 43) at entry into TED12037 are shown in Table 2. There were only minor differences between the baseline characteristics of the all-treated and BMF analysis populations. Demographic data for the 59 patients who started in TED12037 have been published previously [24].

**Table 2:** Baseline characteristics of BMF analysis and all-treated populations at entry into TED12037

Characteristic	BMF analysis population (n = 21)	All-treated population (n = 43)
Mean age, years (SD)	63.5 (10.7)	63.8 (10)
Sex, n (%)		
Male	12 (57)	26 (60)
Female	9 (43)	17 (40)
Disease subtype, n (%)		
PMF	12 (57)	30 (70)
Post-ET MF	1 (5)	2 (5)
Post-PV MF	8 (38)	11 (26)
Risk status [27], n (%)		
High	8 (38)	15 (35)
Intermediate	13 (62)	28 (65)
*JAK2*V617F mutation status, n (%)		
Positive	19 (90)	39 (91)
Negative	2 (10)	4 (9)
Last fedratinib dose in TED12037, mg/day		
120	3 (14)	3 (7)
240	2 (10)	4 (9)
360	1 (5)	5 (12)
400	0	1 (2)
440	2 (10)	4 (9)
520	7 (33)	11 (26)
600	1 (5)	3 (7)
680	5 (24)	12 (28)
BMF grade, n (%)		
0	0 (0)	0 (0)
1	2 (10)	2 (5)
2	10 (48)	10 (23)
3	9 (43)	10 (23)^a,b^
Spleen size (cm), median (range)	18 (4–34)	18 (4–34)
Haemoglobin (g/dL), median (range)	9.7 (7.4–15.2)	9.3 (7.3–15.2)
White blood cells (×10^9^/L), median (range)	16.4 (2.1–103.3)	14.9 (2.1–103.3)

Percentages may not sum to 100 due to rounding

^a^One patient had a baseline BMF reading but no subsequent BMF readings and was therefore excluded from the BMF population

^b^Twenty-one patients had missing baseline BMF data

Patients were receiving fedratinib doses ranging from 120 to 680 mg/day at the end of TED12037 and continued on these doses upon entry to TED12015. The overall median fedratinib dose across both studies was 489.6 mg/day (range 144.5–682.7) and the median treatment duration was 35 cycles (range 7–61).

### Bone marrow fibrosis

**Fig. 1:**
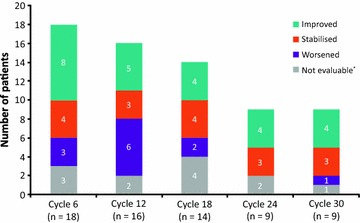
Summary of bone marrow fibrosis changes from baseline, by treatment cycle (*Persistent grade 3)

A total of 153 bone marrow samples were analysed (including post-baseline samples from patients excluded from the BMF population due to missing baseline data), of which 87 samples were included in the analysis reported herein. A consensus on BMF grade was reached without discussion for 150 (98 %) of the samples, with agreement among all three haematopathologists for 118 (77 %) and between two pathologists for 32 (21 %) of the samples. BMF grade for the remaining three samples (2 %) was assigned following open discussion, in accordance with the central pathology review charter. Changes in BMF grade from baseline for each patient in the BMF population are summarised in Table 3. Figure 1 illustrates the proportion of patients with improvement, stabilisation or worsening of BMF according to treatment cycle. Because of the small numbers of patients at later time points, proportional data are presented up to cycle 30. Of the nine patients evaluable at cycle 30, four (44 %) had shown an improvement in BMF grade and four (44 %) had shown no change in BMF grade. Two of the patients with BMF improvement at cycle 30 had a two-grade fibrosis reduction from their respective baselines.

**Table 3:** Changes in BMF grade from baseline for individual patients, according to disease subtype and treatment cycle

Disease subtype	BMF grade						Change in BMF grade from baseline to last time point evaluated
	Baseline (n = 21)	Cycle 6 (n = 18)	Cycle 12 (n = 16)	Cycle 18 (n = 14)	Cycle 24 (n = 9)	Cycle 30 (n = 9)	
PMF	3	2	n/a	n/a	n/a	n/a	Improved
PMF	3	2	1	1	n/a	1	Improved
PMF	3	3	n/a	3	n/a	n/a	Persistent grade 3
PMF	3	3	n/a	n/a	n/a	n/a	Persistent grade 3
PMF	3	2	3	n/a	2	2	Improved
PMF	3	2	2	2	2	n/a	Improved
PMF	2	3	3	n/a	n/a	n/a	Worsened
PMF	2	2	2	2	n/a	n/a	Stabilised
PMF	2	n/a	3	n/a	n/a	n/a	Worsened
PMF	2	n/a	3	n/a	n/a	n/a	Worsened
PMF	1	1	2	1	1	1	Stabilised
PMF	1	n/a	0	0	0	n/a	Improved
Post-PV MF	3	2	2	3	n/a	n/a	Persistent grade 3
Post-PV MF	3	3	n/a	3	3	3	Persistent grade 3
Post-PV MF	3	2	3	3	3	n/a	Persistent grade 3
Post-PV MF	2	3	2	2	n/a	2	Stabilised
Post-PV MF	2	1	0	0	n/a	0	Improved
Post-PV MF	2	3	3	3	2	3	Worsened
Post-PV MF	2	2	3	n/a	2	2	Stabilised
Post-PV MF	2	2	2	2	1	1	Improved
Post-ET MF	2	1	n/a	3	n/a	n/a	Worsened

*n/a* not applicable

Changes in BMF according to the baseline characteristics and overall clinical response of individual patients are listed in additional file 1: Table S1. In some cases, improvements in BMF coincided with a reduction in palpable splenomegaly with fedratinib dosing beyond cycle 6. Many patients in the BMF analysis population achieved either stable disease or clinical improvement, as assessed by the IMWG-MRT criteria.

**Fig. 2:**
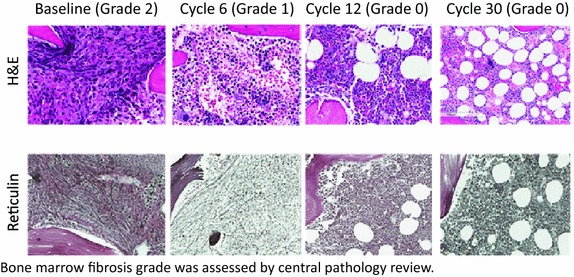
Representative histological images for Patient 1 showing complete resolution of bone marrow fibrosis (grade 2 to grade 0)

**Fig. 3:**
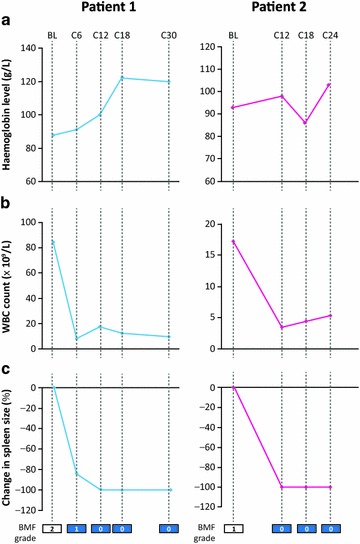
Clinical assessments in patients with complete resolution of bone marrow fibrosis. **a** Haemoglobin levels, **b** white blood cell count and **c** change in spleen size

Two patients had achieved complete resolution of fibrosis (BMF grade 0) by treatment cycle 12. Patient 1, with post-PV MF, presented with a spleen size of 13 cm and BMF grade of 2 at baseline. Patient 2, with PMF, presented with a spleen size of 4 cm and BMF grade 1 at baseline. Patient 1 achieved complete clinical remission, according to IWG-MRT response criteria, starting at cycle 24 and continuing to cycle 40. Figure 2 shows representative images for Patient 1 at different treatment cycles. In both patients, white blood cell counts normalised during treatment and a reduction in spleen size of up to 100 % was recorded. Haemoglobin levels normalised in patient 1 and improved in patient 2. Haematological and clinical assessments for these two patients are shown in Fig. 3. Scatter plots depicting the distribution of WBC levels, spleen size changes, and haemoglobin levels in individual patients at each treatment cycle based on BMF status are shown in additional file 2: Figure S1.

## Discussion

Identification of the high frequency of activating mutations affecting *JAK/STAT* signalling in patients with Ph-negative (Ph−) MPNs has established dysregulation of the *JAK/STAT* signalling pathway as the major contributor to the pathogenesis of haematopoietic stem and progenitor cell derived MPNs [3]. In patients with MPNs, aberrant signalling from the neoplastic haematopoietic clone results in increased levels of inflammatory and angiogenic cytokines, as well as fibrotic changes in the bone marrow stroma, causing the clinical signs and symptoms seen in myelofibrosis [1, 30]. A direct causal role for enhanced *JAK2* signaling in bone marrow fibrosis has been demonstrated in mice transplanted with *JAK*2V617F+ bone marrow cells [23]. The underlying pathogenic mechanisms in MPNs still require further elucidation; however, our current understanding supports the rationale for inhibition of *JAK* signalling with anti-*JAK1* and anti-*JAK2* agents that could potentially inhibit the inflammatory signalling cascade, preventing or reducing BMF in patients with Ph− MPNs.

The results of this exploratory analysis support this hypothesis by demonstrating that prolonged treatment with the *JAK2* inhibitor, fedratinib, was associated with stabilisation or improvement of BMF in a proportion of patients with PMF, post-ET myelofibrosis and post-PV myelofibrosis. These findings are in agreement with recently published data from studies of ruxolitinib therapy, which also suggested that *JAK* inhibition may prevent the progression of BMF in some patients with MPNs with myelofibrosis [20, 21]. Although the small number of patients studied precluded statistical comparisons, the results suggest that improvement in BMF may be associated with concomitant improvements in splenomegaly and peripheral blood counts in some patients. The generalizability of our results is clearly limited by the small sample size and by the inherent bias associated with focusing the analyses on patients who were responding to long-term fedratinib treatment (15 of the 59 patients who began the phase 1 study discontinued treatment early).

In this era of disease-modifying agents, such as *JAK2* and *JAK1/JAK2* inhibitors, accurate grading of BMF has become crucial not only for the precise diagnosis of MPNs, but also for assessment of response to treatment. This study used the BMF grading system advocated by the World Health Organization 2008 classification, which is based on semi-quantitative assessment of reticulin stain on a 4-grade scale (from 0 to 3) (2008 WHO Classification of Tumors of Hematopoietic and Lymphoid Tissue) [14]. This study showed a high level of agreement on reticulin fibrosis grading among the three pathologists (98 %), when using this system [31]. However, these criteria do have limitations, in particular when evaluating the dynamic changes of BMF associated with greater tissue heterogeneity following treatment with disease-modifying agents; further study is needed to optimise BMF grading in the setting of this post-treatment heterogeneity, which is not addressed in the current WHO grading system [31]. Despite these limitations, use of BMF grading has previously been demonstrated to have diagnostic and prognostic value in patients with Ph− MPNs [13–15], and it is therefore important that the effects of novel therapies on BMF are adequately investigated and reported.

Current practice in the management of myelofibrosis is to use a range of clinical and demographic parameters to risk-categorise patients [32]. Recent studies suggest that the addition of BMF grading to the International Prognostic Scoring System improves prognostication of patients with PMF [14]. Patients with PV and fibrosis at presentation are less prone to experience thrombosis during their clinical course and more prone to develop post-PV MF [33]. These results, in conjunction with our analysis, add support to the importance of including BMF assessment at presentation and during the treatment of patients with myelofibrosis.

## Conclusion

These findings with a *JAK2* selective inhibitor, fedratinib, together with the previously reported effects of the *JAK1/JAK2* inhibitor ruxolitinib, indicate that improvement or even resolution of BMF in some patients with myelofibrosis may be achievable with JAK2 inhibitor therapy.

## Additional files

Additional file 1:
**Table S1.** Changes in BMF according to baseline characteristics and clinical response. Additional file 2:
**Figure S1.** Scatter plots depicting the distribution of WBC levels (A), spleen size changes (B), and haemoglobin levels (C) in individual patients at each treatment cycle based on BMF status.

## Abbreviations

BL: baseline
BMF: bone marrow fibrosis
C: cycle
ET: essential thrombocythaemia
H&E: haematoxylin and eosin
MF: myelofibrosis
PMF: primary myelofibrosis
PV: polycythaemia vera
SD: standard deviation
WBC: white blood cell
