# Supplementary material for: Effect of treatment with a JAK2-selective inhibitor, fedratinib, on bone marrow fibrosis in patients with myelofibrosis
Source: J Transl Med. 2015 Sep 10;13:294. doi: 10.1186/s12967-015-0644-4 (PMC4566296; doi:10.1186/s12967-015-0644-4)
Supplement: Additional file 1: — Table S1. Changes in BMF according to baseline characteristics and clinical response. [file 12967_2015_644_MOESM1_ESM.docx]

**Table S1: Changes in BMF according to baseline characteristics and clinical response***

| **Patient** | **Overall change in BMF grade from baseline** | **Diagnosis** | **Risk category** | **Spleen size (cm)** | **JAK2V617F** | **Last dose in TED12037 (mg/day)** | **Average dose (mg/day)** | **Total exposure (cycles)** | **Clinical response (spleen volume)** |
| --- | --- | --- | --- | --- | --- | --- | --- | --- | --- |
| 1 | Improved (2→0 [C12]) | Post-PV MF | High | 13 | Positive | 680 | 486 | 52 | Improved |
| 2 | Improved (1→0 [C12]) | PMF | Intermediate | 4 | Negative | 120 | 410 | 33 | Stable disease |
| 3 | Improved (3→1 [C12]) | PMF | Intermediate | 14 | Positive | 520 | 476 | 34 | Improved |
| 4 | Improved (2→1 [C24]) | Post-PV MF | High | 6 | Positive | 520 | 513 | 46 | Improved |
| 5 | Improved (3→2 [C12]) | PMF | Intermediate | 13 | Positive | 240 | 144 | 7 | Stable disease |
| 6 | Improved (3→2 [C24]) | PMF | High | 19 | Positive | 680 | 686 | 34 | Stable disease |
| 7 | Improved (3→2 [C6]) | PMF | Intermediate | 15 | Positive | 440 | 363 | 48 | Improved |
| 8 | Stabilised (G1) | PMF | Intermediate | 11 | Positive | 120 | 379 | 61 | Stable disease |
| 9 | Stabilised (G2) | PMF | High | 25 | Positive | 520 | 518 | 47 | Improved |
| 10 | Stabilised (G2) | Post-PV MF | Intermediate | 16 | Positive | 120 | 390 | 49 | Stable disease |
| 11 | Stabilised (G2) | Post-PV MF | High | 34 | Positive | 680 | 529 | 54 | Improved |
| 12 | Persistent (G3) | PMF | Intermediate | 18 | Negative | 600 | 602 | 45 | Stable disease |
| 13 | Persistent (G3) | PMF | Intermediate | 23 | Positive | 680 | 673 | 13 | Stable disease |
| 14 | Persistent (G3) | Post-PV MF | Intermediate | 26 | Positive | 520 | 462 | 26 | Stable disease |
| 15 | Persistent (G3) | Post-PV MF | Intermediate | 18 | Positive | 520 | 618 | 45 | Stable disease |
| 16 | Persistent (G3) | Post-PV MF | High | 23 | Positive | 440 | 413 | 35 | Stable disease |
| 17 | Worsened | PMF | Intermediate | 27 | Positive | 520 | 461 | 9 | Relapsed following early improvement |
| 18 | Worsened | PMF | High | 10 | Positive | 360 | 317 | 12 | Improved |
| 19 | Worsened | PMF | Intermediate | 25 | Positive | 680 | 611 | 19 | Stable disease |
| 20 | Worsened | Post-PV MF | Intermediate | 17 | Positive | 240 | 444 | 58 | Improved |
| 21 | Worsened | Post-ET MF | High | 18 | Positive | 520 | 539 | 17 | Improved |
| *Greyed rows indicate patients with improvements in both BMF and spleen volume. | | | | | | | | | |
