# Supplementary material for: Effect of treatment with a JAK2-selective inhibitor, fedratinib, on bone marrow fibrosis in patients with myelofibrosis
Source: J Transl Med. 2015 Sep 10;13:294. doi: 10.1186/s12967-015-0644-4 (PMC4566296; doi:10.1186/s12967-015-0644-4)
Supplement: Additional file 2: — Figure S1. Scatter plots depicting the distribution of WBC levels (A), spleen size changes (B), and haemoglobin levels (C) in individual patients at each treatment cycle based on BMF status. [file 12967_2015_644_MOESM2_ESM.pptx]

## Slide 1
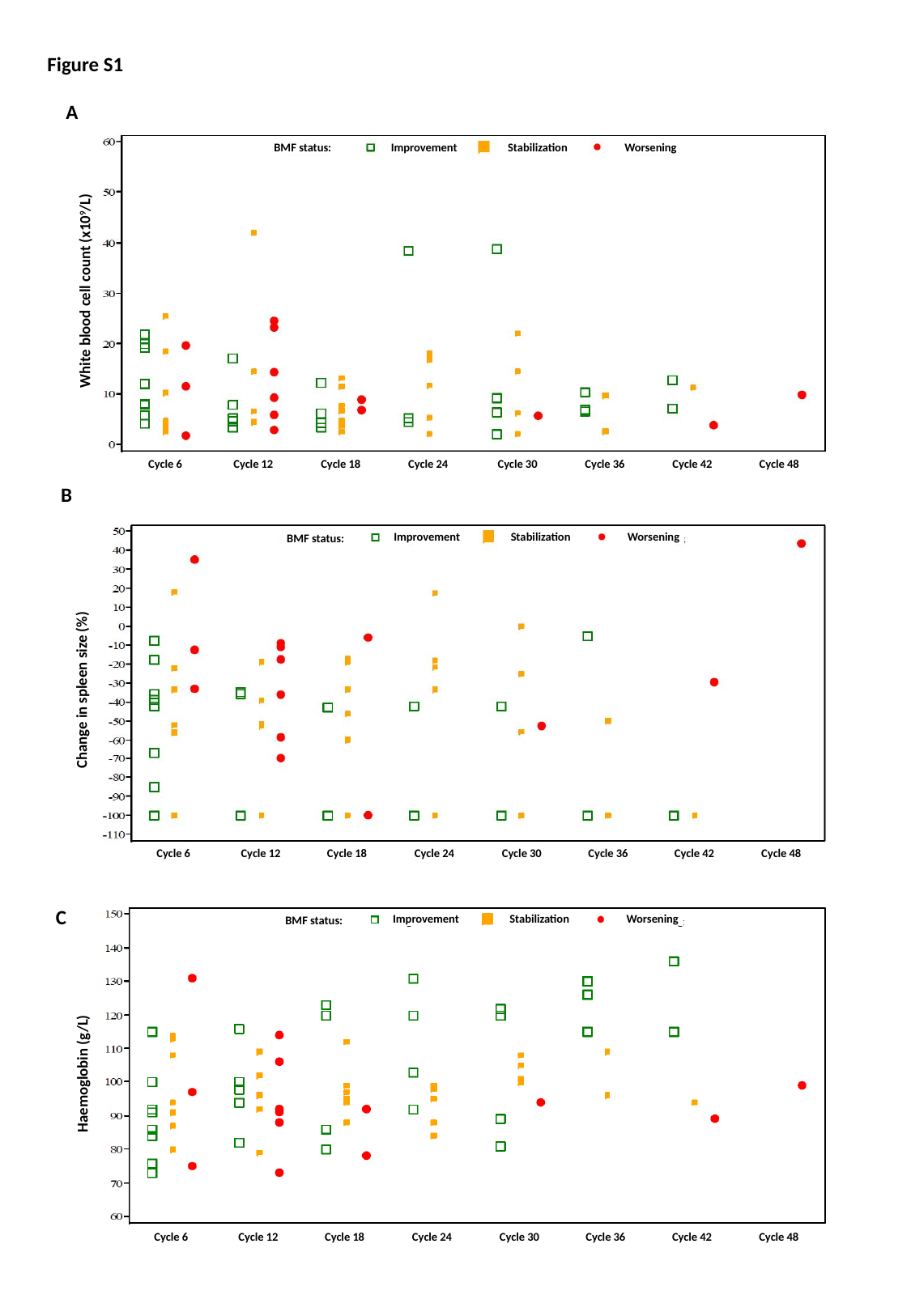

Figure S1
A
White blood cell count (x109/L)
Worsening
Stabilization
Improvement
BMF status:
Cycle 6
Cycle 12
Cycle 18
Cycle 24
Cycle 30
Cycle 36
Cycle 42
Cycle 48
B
Worsening
Stabilization
Improvement
BMF status:
Change in spleen size (%)
Cycle 6
Cycle 12
Cycle 18
Cycle 24
Cycle 30
Cycle 36
Cycle 42
Cycle 48
C
Worsening
Stabilization
Improvement
BMF status:
Haemoglobin (g/L)
Cycle 6
Cycle 12
Cycle 18
Cycle 24
Cycle 30
Cycle 36
Cycle 42
Cycle 48
